# Biodegradable and Elastomeric Poly(glycerol sebacate) as a Coating Material for Nitinol Bare Stent

**DOI:** 10.1155/2014/956952

**Published:** 2014-05-13

**Authors:** Min Ji Kim, Moon Young Hwang, JiHeung Kim, Dong June Chung

**Affiliations:** ^1^Department of Polymer Science and Engineering, Sungkyunkwan University, Suwon 440746, Republic of Korea; ^2^School of Chemical Engineering, Sungkyunkwan University, Suwon 440746, Republic of Korea

## Abstract

We synthesized and evaluated biodegradable and elastomeric polyesters (poly(glycerol sebacate) (PGS)) using polycondensation between glycerol and sebacic acid to form a cross-linked network structure without using exogenous catalysts. Synthesized materials possess good mechanical properties, elasticity, and surface erosion biodegradation behavior. The tensile strength of the PGS was as high as 0.28 ± 0.004 MPa, and Young's modulus was 0.122 ± 0.0003 MPa. Elongation was as high as 237.8 ± 0.64%, and repeated elongation behavior was also observed to at least three times the original length without rupture. The water-in-air contact angles of the PGS surfaces were about 60°. We also analyzed the properties of an electrospray coating of biodegradable PGS on a nitinol stent for the purpose of enhancing long-term patency for the therapeutic treatment of varicose veins disease. The surface morphology and thickness of coating layer could be controlled by adjusting the electrospraying conditions and solution parameters.

## 1. Introduction

Biodegradable elastomers are important materials for a wide variety of medical applications. Elastomers have gained popularity because they can provide stability and structural integrity in mechanically dynamic environments without irritation to the host tissues [1, 2], and they exhibit mechanical properties similar to those of soft tissues [3–5]. For the special scaffold requiring strong mechanical properties, tough and biodegradable elastomers (poly(*ε*-caprolactone) (PCL) [6, 7], poly(glycerol sebacate) (PGS) [8], and their blended materials) were frequently adapted for* in vivo* tissue regeneration or substitution trials in many clinical fields [9–11]. But these attempts were mainly related to the improvement of tissue regeneration capability, drug sustainability, and cell adhesion properties through electrospinning process [12–14].

Stent surgery is widely used for therapeutic treatment of coronary artery and varicose veins disease, and several kinds of commercialized bare metal stents have been used in clinical settings in spite of their risks including inflammation, late thrombosis or restenosis, and fracture formation in long-term duration.

To reduce such complications, we selected PGS as biodegradable and elastic polymer for the enhancement of mechanical strength and durability of bare nitinol stent which is used for the interventional treatment of superficial femoral artery disease. PGS (one of the excellent, tough, and biodegradable polymers) was obtained as low molecular weight (<10,000) prepolymer through polycondensation and often blended with PCL for satisfying electrospray condition because of its weak solution property (low viscosity in organic solvent). After electrospray coating, their own tough and elastic behaviors were exhibited through additional curing reaction.

In this study, we synthesized relatively high molecular weight (31,000 in *M*_*w*_) PGS prepolymer and examined the suitability for electrospray coating method (a useful technique to obtain uniform coating layer on three-dimensional structures [15–20]), and we also confirmed the biodegradable and elastomeric properties after postcuring. For the basic study of this purpose, we measured the surface morphology and thickness of the coated films to study the feasibility of PGS as a novel coating material for nitinol bare stent. By this method, we expect that stent durability, which is essential factor for the therapeutic treatment of varicose veins disease, will enhance.

## 2. Experimental

### 2.1. Materials and Synthesis of PGS [2, 8]

The PGS polymer was synthesized by polycondensation of 0.1 mol each of glycerol (glycerin, 99.0%, Samchun Pure Chemical Co., Seoul, Korea) and sebacic acid (Tokyo Chem. Indus. Co., Tokyo, Japan). Both reagents were mixed together in a three-necked flask at 130°C under an argon environment for 3 h, and the pressure of reaction flask was reduced from 1 Torr to 40 mTorr. After pressure reduction, the reaction was continued for 45 h at 120°C under a reduced atmosphere. Then partly cross-linked PGS prepolymer was obtained (yield for viscous liquid phase polymer, above 80%). Supplemental cross-linking (postcuring) reaction of PGS prepolymer was done in vacuum oven at 100°C for additional 48 hours.

### 2.2. Polymer Characterization

The resulting material after supplemental cross-linking was soaked in 100% ethanol for 24 h and was subsequently soaked in PBS for 24 h to remove unreacted reagents prior to instrumental analysis and mechanical testing.

Polymer synthesis was confirmed by GPC, ^1^H-NMR, and FT-IR measurements. GPC (gel permeation chromatography, Waters 515, Styragel column, Milford, MA, USA) was used to measure the time-dependent molecular weight changes of PGS prepolymer. A PGS solution (1 wt%) was prepared in chloroform (as the mobile phase of GPC) for the measurements. The elution rate of the mobile phase was adjusted to 1 mL/min, and a styrene standard was used for molecular weight calibration. ^1^H-NMR (nuclear magnetic resonance, Varian Unity Inova, 500 MHz, Germany) spectra for PGS prepolymer were obtained using CDCl_3_ as a solvent The chemical composition was determined by calculating the signal integrals of –COCH_2_CH_2_CH_2_– at 1.2, 1.5, and 2.2 ppm for sebacic acid and –CH_2_CH– at 3.7, 4.2, and 5.2 ppm for glycerol. Also, each functional group of the synthesized polymers was examined by FT-IR (Bruker IFS-66/S FT-IR, Bruker Optics, Germany).

Tensile tests after postcuring were conducted on 22 × 6 × 1.5 mm (according to ASTM standard D412-a) polymer strips cut from polymer sheets on a UTM LR30K Plus (Lyord Instrument Ltd., West Success, UK) equipped with a 250 N load cell.

The strain rate was 50 mm/min, and all samples were elongated to failure. Values were converted to stress-strain and Young's modulus was calculated from the initial slope using 4–6 samples. The cross-linking density (*n*) was calculated according to the theory of rubber elasticity using the following equation [2, 21]:

(1) $$\begin{array}{c} n=\;\frac{E_{0}}{3RT\;}=\;\frac{\rho }{{\left(M_{w}\right)}_{c}}, \end{array}$$

where *n* represents the number of active network chain segments per unit volume (mol/m^3^), (*M*_*w*_)_*c*_ represents the molecular weight between cross-links (g/mol), *E*_0_ represents Young's modulus (Pa), *R* is the universal gas constant, *T* is the absolute temperature (K), and *ρ* is the measured elastomer density (g/cm).

Swelling by hydration of postcured PGS was conducted by the immersion of cross-linked PGS samples in PBS (phosphate buffered solution), deionized water, and ethanol. The swelling ratio was calculated using the following equation:

(2) $$\begin{array}{c} \text{Swelling}\;\text{ratio}\;\left(\%\right)=\;\frac{W_{s}-W_{o}}{W_{o}}\times 100, \end{array}$$

where *W*_*s*_ represents the weight of swollen PGS and *W*_*o*_ represents the weight of dried PGS.

To calculate the surface energy of the polymers, we measured the contact angles in deionized water, dodecane, 1,1,2,2-tetrabromoethane, and glycerin using a contact angle meter (GBX DIGIDROP, Scientific Instrumentation, Romans, France) at room temperature. The surface energy was calculated by the following:

(3) $$\begin{array}{c} \gamma_{s}=\gamma_{s}^{a}+\gamma_{s}^{b}+\gamma_{s}^{c},\\ \gamma \left(1+\cos\theta \right)=2\sqrt{\gamma_{s}^{a}\cdot \gamma_{L}^{a}}+2\sqrt{\gamma_{s}^{b}\cdot \gamma_{L}^{b}}\;+2\sqrt{\gamma_{s}^{c}\cdot \gamma_{L}^{c}}, \end{array}$$

where *γ*_*s*_^*a*^, *γ*_*s*_^*b*^, *γ*_*s*_^*c*^ were collected from a previous report [22].

### 2.3. *In Vitro* Degradation

The degradation test was conducted using an enzyme solution (porcine liver esterase, 40 units/mL in PBS), an NaOH solution (0.1 mM), and PBS (pH 7.0). Disk-type postcured PGS specimens (10 mm in diameter, 2 mm in thickness) were degraded under* in vitro* conditions for predetermined time intervals. Degradation profiles were measured by incubating PGS in three kinds of solutions (20 mL) at 37°C with shaking. After the predetermined incubation time, the samples were removed, washed in deionized water, dried at 90°C for seven days, and weighed to determine the weight loss. The degradation ratio was calculated by comparing the initial weight (*W*_0_) with the weight measured at a given time point (*W*_*t*_).

### 2.4. Electrospray Coating of PGS on Nitinol Stents

**Figure 1:**
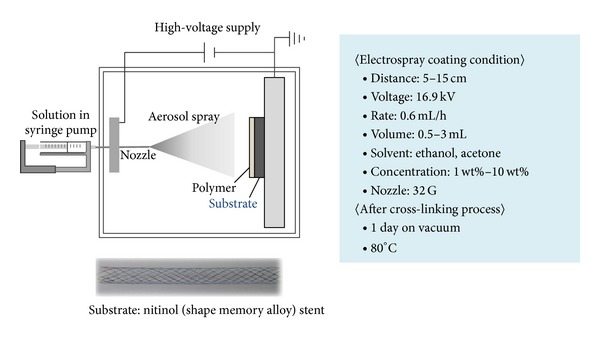
Schematic diagram of the electrospray coating process.

Nitinol stents (10 × 75 mm, diameter × length) were kindly provided from S&G Bio Co. (Sungnam, Korea) and used as specimens in the PGS prepolymer coating experiments. They were cleaned before use in a water/ethanol (1 : 1 in volume ratio) solution using an ultrasonic cleaner. The experimental setup and processing conditions for the electrospray coating are shown in Figure 1. An acetone and ethanol mixture (3 : 7 in volume ratio) was used as a solvent for the PGS prepolymer (*M*_*w*_ = 31,000 g/mol) solution. The electrospray coating process was done using eSpray electrospraying system (NanoNC, Seoul, Korea) equipped with 20 mL syringe fitted with a needle (32 gauge, I.D., 0.1 mm and O.D., 0.23 mm) on KDS 100 syringe pump (KD Science, Holliston, MA, USA). Nitinol stent was rotating on the collector side connected with electrode during electrospray coating process. After electrospray coating, for the supplemental cross-linking of coated PGS, the coated stents were placed in a vacuum oven at 100°C for 48 hrs additionally.

The morphologies of the coated surfaces were characterized by scanning electron microscopy (SEM; S-2400, Hitachi, Tokyo, Japan). The polymer-coated surfaces were sputter-coated with Au-Pd using a sputtering system before SEM observation. To evaluate the thickness of the coated polymer films on the stent strut, the morphology of the cross-sectionally cut surface in a liquid nitrogen environment was investigated with SEM.

## 3. Results and Discussion

### 3.1. Polymer Characterization

**Figure 2:**
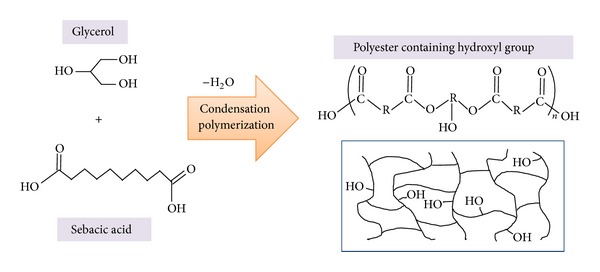
Polycondensation of glycerol and sebacic acid yielding the PGS polymer.

The PGS polymer was prepared by polycondensation of glycerol and sebacic acid (Figure 2) after 48 hours of reaction time. The resulting polymer had a small number of cross-linking points and hydroxyl groups directly attached to the backbone, as were seen by spectroscopic analysis. After 48 hours of reaction time, the obtained partly cross-linked PGS prepolymer had a weight-average molecular weight (*M*_*w*_) of 31,000 and a number average molecular weight (*M*_*n*_) of 2,300 (as determined by GPC) with polydispersity index (PDI) of 13.0. The molar composition of the PGS prepolymer was approximately 1 : 1 glycerol/sebacic acid, as confirmed by ^1^H NMR analysis (proton peaks of –COCH_2_CH_2_CH_2_– shown at 1.2, 1.5, and 2.2 ppm and proton peaks of –CH_2_CH shown at 3.1, 4.2, and 5.2 ppm, data not shown). In addition, we observed FT-IR peaks at 1800–1600 cm^−1^ (C=O), 3500–3200 cm^−1^ (–OH), and 1375 cm^−1^ (–CH) (data not shown), which confirms the existence of ester groups formed through polycondensation.

Such partly cross-linked PGS prepolymer (viscous liquid phase) can be solved in polar organic solvent owing to the remained –OH groups in PGS and applied for stent coating material. But fully cross-linked PGS polymer after postcuring could not dissolve in any kinds of organic solvent and shows elastic behaviors.

### 3.2. Mechanical Tests and Swelling Ratio

**Figure 3:**
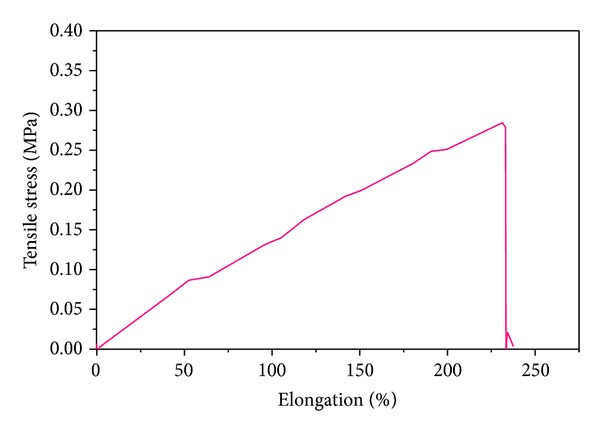
Stress-strain curves of PGS after postcuring for additional 48 h at 100°C.

Tensile test results of thin strips of fully cross-linked PGS through supplemental cross-linking process revealed a stress-strain curve characteristic originating from elastomeric and tough materials (Figure 3). Permanent deformation was not observed during the tensile tests. Young's modulus and elongation at break of the PGS were 0.122 ± 0.0003 MPa and 237.8 ± 0.64% (those of PCL were 225 ± 11 MPa and 93 ± 9% in membrane type [23]). In another report, Young's modulus of PGS:PCL blended membrane is also increased according to the increase of PCL ratio in composite [11]. These data meant that PGS showed more elastic behavior than that of PCL, and the PGS could be elongated repeatedly to several times its original length without rupture. The ultimate tensile strength is greater than 0.3 MPa. The value of Young's modulus of PGS is located between that of ligaments (in KPa range) and tendons (in GPa range), and the strain to failure of PGS is similar to that of arteries and veins (over 260% elongation).

Also, the cross-linking density (*n*) and relative molecular mass between cross-links ((*M*_*w*_)_*c*_) were calculated using the density and Young's modulus of the samples as previously described (see (1)). The cross-linking density was 16.4 mol/m^3^, and the relative molecular mass between cross-links was about 58,000 g/mol.

**Figure 4:**
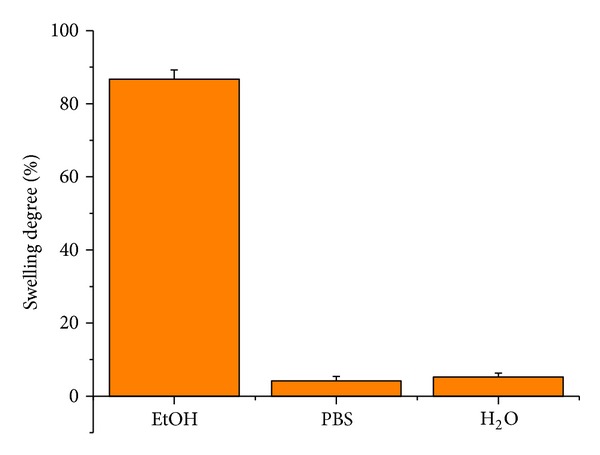
The solvent-dependent swelling behaviors of PGS.

The degree of swelling of the elastomeric networks in ethanol was about 85% (Figure 4). However, the degree of swelling of PGS in deionized water and PBS was about 5%. Therefore, the synthesized polymer should not excessively swell in* in vivo* conditions.

### 3.3. Contact Angle Measurements

Interfacial characteristics of coating polymers are significantly important for films, coating, printing, and adhesives. Water-in-air contact angle of coated PGS surface is about 60° (that of PCL is 120° [9]), and such hydrophilicity of PGS is related to the remained –OH groups after postcuring as shown in Figure 2. This is greatly related to the formation of H bonding between PGS and metal and also affected the adhesion property of PGS on metal stent. In addition, we calculated the surface energy using (3) of the three-component system for the surface tension method with dodecane (a nonpolar solvent), 1,1,2,2-tetrabromoethane (a polar solvent), and glycerin (a hydrogen-bonding solvent). The surface energy of the PGS polymer was 63.13 dyne/cm. This value is relatively high compared to those of polytetrafluoroethylene (19.1 dyne/cm) and polyethylene (33.1 dyne/cm).

### 3.4. *In Vitro* Degradation Studies

**Figure 5:**
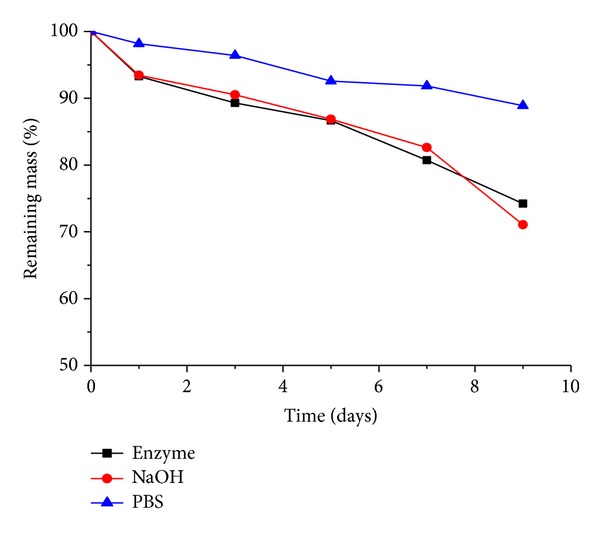
Degradation studies of PGS in solvents (▲: PBS, ●: 0.1 mM NaOH, ■: enzyme solution) at 37°C.

We examined the degradation characteristics of PGS under* in vitro* conditions (Figure 5). Agitation for nine days in NaOH solution at 37°C caused the polymer to degrade by 30%, as measured by change of a dry sample. In enzyme degradation, the PGS polymer was degraded by 25% over nine days in esterase solutions, while it degraded by 10% in PBS solutions.

### 3.5. Electrospray Coating on a Nitinol Stent

**Figure 6:**
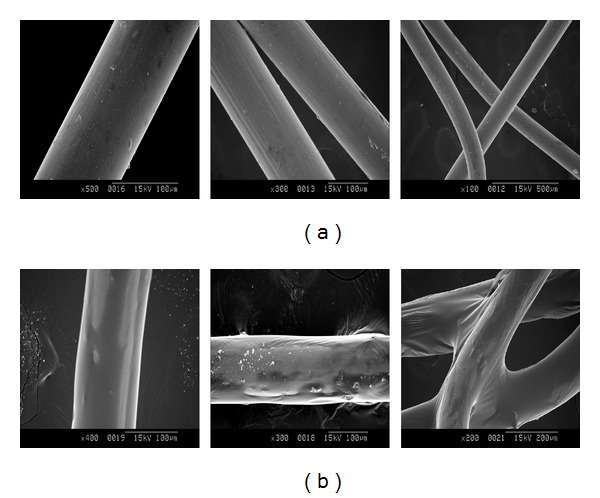
Surface morphology observation of PGS-coated stent by SEM: (a) an uncoated nitinol stent and (b) a PGS-coated stent under the electrospinning conditions of 10 wt% PGS solution in acetone and ethanol mixture (3 : 7 in volume ratio) at 0.6 mL/h flow rate.

The surface morphologies of bare and coated stents were observed using SEM (Figure 6). Compared to bare stents, coated stents had smoother surfaces. Also, we observed the concentration effects of the coating materials on the resulting surface morphologies and thickness.

**Figure 7:**
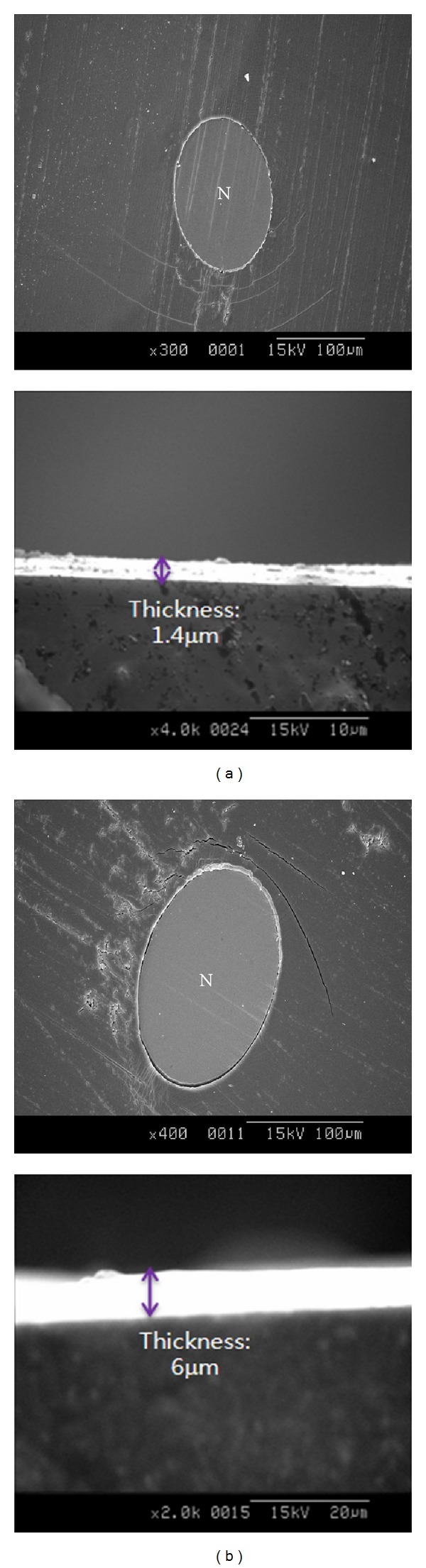
Cross-sectional SEM images of PGS coated on stent struts with volume differences: 1 mL of 1 wt% PGS solution (a) and 10 wt% PGS solution (b) in acetone and ethanol mixture (3 : 7 in volume ratio).

From the microscopic images of cross-sectional area of stent strut as shown in Figure 7, PGS polymer was well coated over the whole area of strut through electrospray method in spite of concentration difference of PGS solutions. When a 1 mL of PGS concentration (1 wt%) was sprayed, the surface was rough and the thickness of the polymer coating was about 1.4 *μ*m. However, the coated stent with same volume of a PGS concentration (10 wt%) showed a smooth surface and a thickness of about 6.0 *μ*m, likely caused by solvent evaporation during the electrospray coating. In high concentration polymer solutions, the solvent evaporates much less than in low concentration solutions, leaving thick and smooth surfaces. In addition, the coating thickness can be adjusted simply using the law of conservation of mass. This shows that the polymeric droplets were continuously deposited on the polymer film during the electrospraying. Thus, the surface morphologies and thickness can be controlled by changing the concentration and amount of polymer solution.

## 4. Conclusion 

In this study, we synthesized biocompatible and elastomeric biomaterials (PGS, poly (glycerol sebacate)) through condensation polymerization. These polymers exhibit tunable mechanical properties and considerable flexibility. We also studied the degradation characteristics of the PGS polymers. For application to biomedical implants, we examined an electrospray coating of PGS on metal stents. By examining the solution parameters, we confirmed that the surface morphology of the coated film is related to the PGS solution concentration, and the thickness of the film is linearly proportional to the volume and concentration. It is expected that drug-eluting stents coated with a drug and PGS polymers can be applied practically in clinical applications.
